# Devon and Exeter Hospital

**Published:** 1895-03-16

**Authors:** 


### HOSPITAL RE-CONSTRUCTION.

### DEVON AND EXETER HOSPITAL.

As stated in THE HOSPITAL of November 10th, 1894, like most old buildings, the Devon and Exeter Hospital is now found to be ill-adapted to the needs of the increased staff that the modern requirements of a hospital render necessary to its efficiency. The reconstruction of the existing buildings, with the addition of a new wing for the reception of patients, was under discussion at a special court of the governors held the end of last month. It appears from a report of the sub-committee that none of the recommendations of Mr. Turner, the architect, were in effect adopted, but that it was determined to limit the alterations and additions to:—(*a*) a rearrangement of the kitchen, cooking staff, and administration department, including a new lift and accommodation for nurses, probationers, and domestic servants in the main building, at a cost of £2,947; (*b*) put new (?) sanitary appliances throughout the hospital at a cost of £65. [What can be done in this direction in a large building like that at Exeter for £65?]—(*c*) a new wing to be erected at a cost of £6,630, containing 24 beds in each ward, to provide accommodation for the patients displaced by the alterations in the old building. We cannot regard the decision of the governors as satisfactory. It is at best but an imperfect attempt to half do something which the best interests of this institution demand should be done thoroughly and completely. When we inspected the infirmary last October we laid stress upon the importance of erecting turrets in connection with the old wards in which the lavatories and bath-rooms can be placed, and where they will be disconnected from the wards.

Again, the decision not to include the new sanitary towers for the Halford wing in the alterations about to be carried out is a lamentable one, dangerous to the well-being of the patients. We gather that this view was held by the sub-committee, who expressed the opinion that they should be built. The new sanitary towers for the whole building would only cost £1,044, and the opening up of the Halford wing and the necessary heating apparatus would cost but another £480. It is, in our view, of the first importance that this further expenditure of £1,524 should be sanctioned by the committee and governors of the Devon and Exeter Hospital with all possible speed. We are surprised that the medical staff should have refrained from protesting against the omission of the new sanitary towers from the present scheme of reconstruction, as these towers are essential to the well-being of the patients, and without them the sanitary condition of the hospital buildings, despite the expenditure about to be incurred, is likely to be unsatisfactory, if it does not even prove highly dangerous to the health and recovery of the patients who occupy it.

